# An updated portrait of monocyte-macrophages in classical Hodgkin lymphoma

**DOI:** 10.3389/fonc.2023.1149616

**Published:** 2023-02-24

**Authors:** Isacco Ferrarini, Andrea Bernardelli, Ester Lovato, Alberto Schena, Mauro Krampera, Carlo Visco

**Affiliations:** Section of Hematology, Department of Medicine, University of Verona, Verona, Italy

**Keywords:** monocytes, macrophages, classical Hodgkin lymphoma, prognosis, microenvironment,

## Abstract

Classical Hodgkin lymphoma (cHL) is a unique neoplastic ecosystem characterized by a heterogeneous immune infiltrate surrounding the rare malignant Hodgkin Reed-Sternberg cells. Though less abundant than T-cells, tumor-infiltrating macrophages play a pivotal role in supporting HRS survival through cell-to-cell and paracrine interactions. Traditional immunohistochemistry based upon the M1-M2 dichotomy yielded controversial results about the composition, functional role and prognostic impact of macrophages in cHL. More recent studies exploiting single-cell technologies and image analyses have highlighted the heterogeneity and the peculiar spatial arrangement of the macrophagic infiltrate, with the most immunosuppressive subpopulations lying in close proximity of HRS cells and the most tumor-hostile subsets kept far away from the neoplastic niches. High-throughput analysis of peripheral blood mononuclear cells in cHL patients have also identified a novel, potentially cytotoxic, subpopulation predicting better response to PD-1 blockade. This review examines the phenotypic profile, spatial localization and clinical impact of tumor-infiltrating macrophages and circulating monocytes in cHL, providing an up-do-date portrait of these innate immune cells with possible translational applications.

## Introduction

Classical Hodgkin lymphoma (cHL) is a B-cell malignancy accounting for 10% of all lymphomas and with a median age at diagnosis of 39.5 years (1). Unlike most human cancers, cHL is characterized by a low number of malignant cells, also known as Hodgkin Reed-Sternberg (HRS) cells, which represent only a minor fraction (1% to 2%) of the overall tumor cellularity (2). HRS cells derive from germinal center B cells, but have lost the expression of several B-cell surface markers, including the B-cell receptor. The genetic hallmark of HRS cells is the 9p24.1 copy gain, which leads to the overexpression of the programmed death-1 (PD-1) axis ligands, *PD-L1* and *PD-L2*. Additionally, *JAK2* is usually included in the 9p24.1 amplicon as well, thus promoting JAK-STAT signaling and further enhancing PD-L1 expression. The major component of the enlarged cHL lymph nodes is an extensive, yet functionally impaired, immune infiltrate including lymphocytes, monocyte-macrophages, NK cells, neutrophils, and eosinophils (3). T-cells are the most abundant immune population in the cHL milieu and comprise a variety of subclusters with different localization patterns and functional properties. T helper (Th) cells are more frequent than cytotoxic T-cells, with PD1^+^ Th1 effector memory and Th1/Treg being the main CD4^+^ subsets expanded in cHL microenvironment (4). These immunosuppressive subpopulations usually lie in the immediate proximity of HRS cells and interact with them through the inhibitory PD-1 or CTLA-4 axes (5, 6). T-cell subsets with potential anti-tumor activity, such as Th17 and CD8^+^ lymphocytes, are restrained outside the neoplastic niches, which are generally defined as an area of 75 μm surrounding the HRS cell (4, 5). Tumor-associated macrophages (TAM) are the second most frequent immune population within the cHL lymph nodes. They are recruited from circulating monocytes by HRS-secreted chemokines, polarize into different functional statuses depending on the local cytokine milieu, and establish an immune network with surrounding T and NK cells that further dampens their cytolytic activity (5, 7, 8). Along the history of monocyte-macrophage research in cHL, three temporal phases can be identified. The first, back to 2010, simply aimed at counting infiltrating macrophages in cHL microenvironment, using CD68 as surface marker (9). The second phase considered the two opposite polarization statuses of human macrophages, i.e., the proinflammatory, anti-tumor M1 subset, and the immunosuppressive, pro-tumor M2 subset, with variable behavior within the cHL tissues (10, 11). Lastly, the third and most recent series of studies went beyond the M1/M2 dichotomy and opened to a new standpoint whereby macrophages can acquire different nuances of functional statuses, express distinct sets of immunoreceptors, and occupy specific areas of the microenvironment, thus contributing to an ordered ecosystem that efficiently supports the growth of HRS cells (5, 12).

### Monocyte subsets and macrophage polarizations

Human monocytes are broadly classified based on their surface expression of CD14 and CD16. CD14^+^CD16^-^ monocytes, also known as classical monocytes, represent more than 80% of the monocyte pool, while the remaining 15-20% includes CD14^+^CD16^+^ intermediate and CD14^low^CD16^+^ non-classical monocytes (13). Classical monocytes are a transient cell population characterized by a range of differentiation potentials. Differently from CD14^low^CD16^+^ monocytes, classical monocytes are equipped with transcriptional programs that allow them to leave the circulation and migrate into tissues under homeostatic conditions. Once released from the bone marrow, classical monocytes circulate in the bloodstream for approximately one day, before extravasating to repopulate a proportion of tissue-resident macrophages or converting into non-classical monocytes (13). Human non-classical monocytes feature a patrolling behavior and efficiently scavenge luminal microparticles as well as monitor the endothelial cell integrity. Under pathological conditions, monocytes gain multiple effector functions including secretion of proinflammatory molecules, antigen-presentation, tissue remodeling, and pro-resolving abilities (13).

TAM are highly plastic cells that derive from both circulating monocytes and tissue-resident macrophages. Historically, macrophages have been functionally clustered into two forms, known as M1 and M2 (14). M1 macrophages can differentiate *in vitro* under the effect of bacterial components and interferons produced during Th1-driven immune responses, whereas M2 polarization is triggered by cytokines released during type 2 immune responses, such as IL-4 and IL-13 (15). M1 state is traditionally associated with macrophage-dependent tissue injury and cancer cell killing, whereas M2 polarization fosters tissue repair and resistance to parasites (15). Surface markers proposed to identify pro-tumoral M2 macrophages are CD206, CD163, and folate receptor-β (16). Although this classification retains a useful communication value, several *in vivo* and *ex vivo* studies have identified macrophage populations with mixed phenotypes, highlighting that the M1/M2 statuses just reflect the extremes of a wide functional spectrum including a variety of intermediate polarizations (17). The cytokine milieu, which can have a distinct composition in different parts of the tissue microenvironment, is responsible for the fine tuning of macrophage polarization throughout the tumor architecture. Similarly, several clinical-grade anticancer drugs, including tyrosine-kinase inhibitors and natural compounds, can modulate the morphological and functional properties of TAM, eventually affecting their phagocytic ability and antigen presentation (18–20).

### Macrophage markers and cHL prognosis: A long-standing debate

The prognostic significance of macrophages in cHL was first shown by Ree and Radin back in 1985. By studying the peanut agglutinin-binding macrophages in 145 cHL patients, they found that a higher number of macrophage-histiocytes was associated with constitutional symptoms (i.e., fever, night sweats, weight loss) and failure to first-line therapy (21). Twenty-five years later, gene expression profiling of 130 cHL diagnostic samples identified a gene signature characterized by the upregulation of TAM and monocytes-related transcripts (9). Such signature was more represented in the group of patients who failed the first-line treatment. Immunohistochemical analysis showed that the extent of CD68^+^ infiltrate correlated with disease-specific survival even in multivariate analysis, outperforming the international prognostic score (IPS). Additionally, the number of TAM correlated with the outcome after second-line treatment (9). Tzankov and colleagues found a direct association of CD68^+^ macrophages with PD-1^+^ and GrB^+^ immune cells within tumor microenvironment, demonstrating that an immunohistochemistry-based score considering all of these three cell types had independent prognostic significance (22). In a subsequent study, the presence of > 25% CD68^+^ macrophages within the cHL tissue defined patients with an inferior 5-year overall survival (OS), retaining prognostic significance in multivariable analysis together with bulky disease and elevated IPS (23). In a phase III trial comparing ABVD and Stanford V chemotherapy in advanced-stage cHL, increased expression of CD68 and CD163 represented an independent predictor of worse failure-free survival and OS (24). Moreover, in this and other works CD68 and CD163 expression correlated with the presence of Epstein-Barr virus (EBV) in neoplastic cells (24, 25). Similarly, a TAM proportion greater than 25% was associated with unfavorable outcome in early-stage cHL cases as well (26). Although this initial series of studies proposed a large macrophage infiltrate as a predictor of poor outcome in cHL patients, additional translational research in the field led to conflicting results. Two studies involving cohorts of 100 and 265 patients, respectively, found no association of CD68^+^ and CD163^+^ macrophage infiltrate with clinical outcome (27, 28). Such discrepancies could be due to the lower sensitivity of immunohistochemical staining to discriminate M1 from M2 macrophages, two subsets with potentially opposite roles within the neoplastic niche. In addition, the concomitant assessment of multiple markers, including PD-L1 and CD86, could be needed to capture the functional status of infiltrating macrophages and more accurately predict clinical outcomes. More recently, a metanalysis of 22 studies, 2959 cHL patients on the whole, showed that high density of either CD68^+^ or CD163^+^ TAM predicted shorter progression-free survival (PFS) and poorer OS, also confirming the association between large macrophage infiltrates and EBV positivity (29). An additional study showed that cHL macrophages expressing MYC, a transcription factor considered as a surrogate marker for M2 polarization, accounted for 21% of all CD68^+^ macrophages (30). In this work, an intermediate number of infiltrating macrophages was associated to better prognosis than very low or very high macrophage density, thus suggesting the “hormesis hypothesis” whereby a certain proportion of macrophages may be beneficial to control the expansion of HRS cells (30). Literature data concerning the prognostic role of macrophages in cHL are summarized in **Table 1**.

**Table 1:** Prognostic impact of tissue macrophages and circulating monocytes in cHL.

Tissue macrophages and cHL prognosis						
Authors	Number of patients	Treatment	Antibodies	Scoring	Threshold	Outcome correlation
Steidl C et al. (8)	166	ABVD ± RT (99%), RT alone (1%)	CD68 (KP1)	Visual estimation	<5% score 1; 5-25% score 2; 25-50% score 3; >50% score 4;	CD68 and PFS/DSS: adverse
Tzankov A et al. (21)	105	ABVD ± RT (28%), COPP ± (46%), RT alone (26%)	CD68 (PGM1)	Visual cell count	<0,82%; >0,82%;	CD68 and OS: adverse
Jakovic LR et al. (22)	52	ABVD ± RT (100%)	CD68 (PGM1)	Visual estimation	<25%; >25%.	CD68 and OS/EFS: adverse
Tan KL et al. (23)	287	ABVD ± RT (50%), Stanford V ± (50%)	CD68 (KP1 and PGM1), CD163 (10D6)	Computer-assisted image analysis	CD68 12,7%; CD163 16,8%	CD68 and FFS/OS: adverse; CD163 and FFS/OS: adverse
Kamper P et al. (24)	288	ABVD/COPP ± RT; ABVD ± RT; RT alone	CD68 (KP1), CD163 (10D6)	Computer-assisted point counting	CD68 7,8%; CD163 21,1%	CD68 and EFS/OS: adverse; CD163 and EFS/OS: adverse
Gotti M et al. (25)	106	ABVD ± RT (100%)	CD68 (PGM1)	Visual estimation	<5% group A; 5-25% group B; 25-50% group C.	CD68 and PFS: adverse
Kayal S et al. (26)	100	ABVD ± RT (88%), EVAP RT (11%), others (1%)	CD68 (CD68/G2)	Visual cell counting	12,9%, 18,2% and 25%	CD68 and PFS/DSS: not associated
Azambuja D et al. (27)	265	ABVD ± RT (100%)	CD68 (KP1), CD163 (10D6)	Visual estimation	<5%, 5-25%, >25%	CD68 and PFS/DSS: not associated; CD163 and PFS/DSS: not associated
Werner L et al. (29)	84	GHLSG guidelines (ABVD and/or BEACOPP± RT) (100%)	CD68 (PG-M1), CD163 (10D6), MYC (EP121)	Computer-assisted image analysis	CD68 <724, 725-937, >938; CD163 <769, 770-1325, >1326	CD68 and DFS/OS: adverse; CD163 and DFS/OS: adverse

Peripheral blood monocytes and cHL prognosis						
Authors	Number of patients	Treatment	Median LMR	LMR cut-off	Outcome correlation	
Koh YW et al. (31)	312	Chemotherapy (67.3%), Radiochemotherapy (32.7%)	2.64	2.9	LMR ≤ 2.9 and EFS/OS: adverse	
Porrata LF et al. (32)	476	Chemotherapy (55%), Radiochemotherapy (45%)	NR	1.1	LMR ≤ 1,1 and LSS/PFS/TTP/OS: adverse	
Jakovic LR et al. (33)	101	Radiochemotherapy (100%)	NR	2	LMR < 2 and EFS/OS: adverse	
Romano A et al. (34)	180	Chemotherapy (46%), Radiochemotherapy (54%)	2.1	2	LMR < 2 and PFS: adverse	

ABVD doxorubicin, bleomycin, vinblastine, dacarbazine, BEACOPP bleomycin, etoposide, doxorubicin, cyclophosphamide, vincristine, procarbazine, and prednisone, COPP cyclophosphamide, vincristine, procarbazine, and prednisone, EVAP etoposide, vinblastine, adriamycin and prednisolone, Stanford V vinblastine, doxorubicin, vincristine, bleomycin, mustard, etoposide, and prednisone, RT radiotherapy, NR not reported, PFS progression-free survival, EFS event-free survival, DSS disease-specific survival, OS overall survival, TTP time to progression, LMR lymphocyte-monocyte ratio.

### Soluble circuitries leading to monocyte recruitment and macrophage polarization in cHL

**Figure 1:**
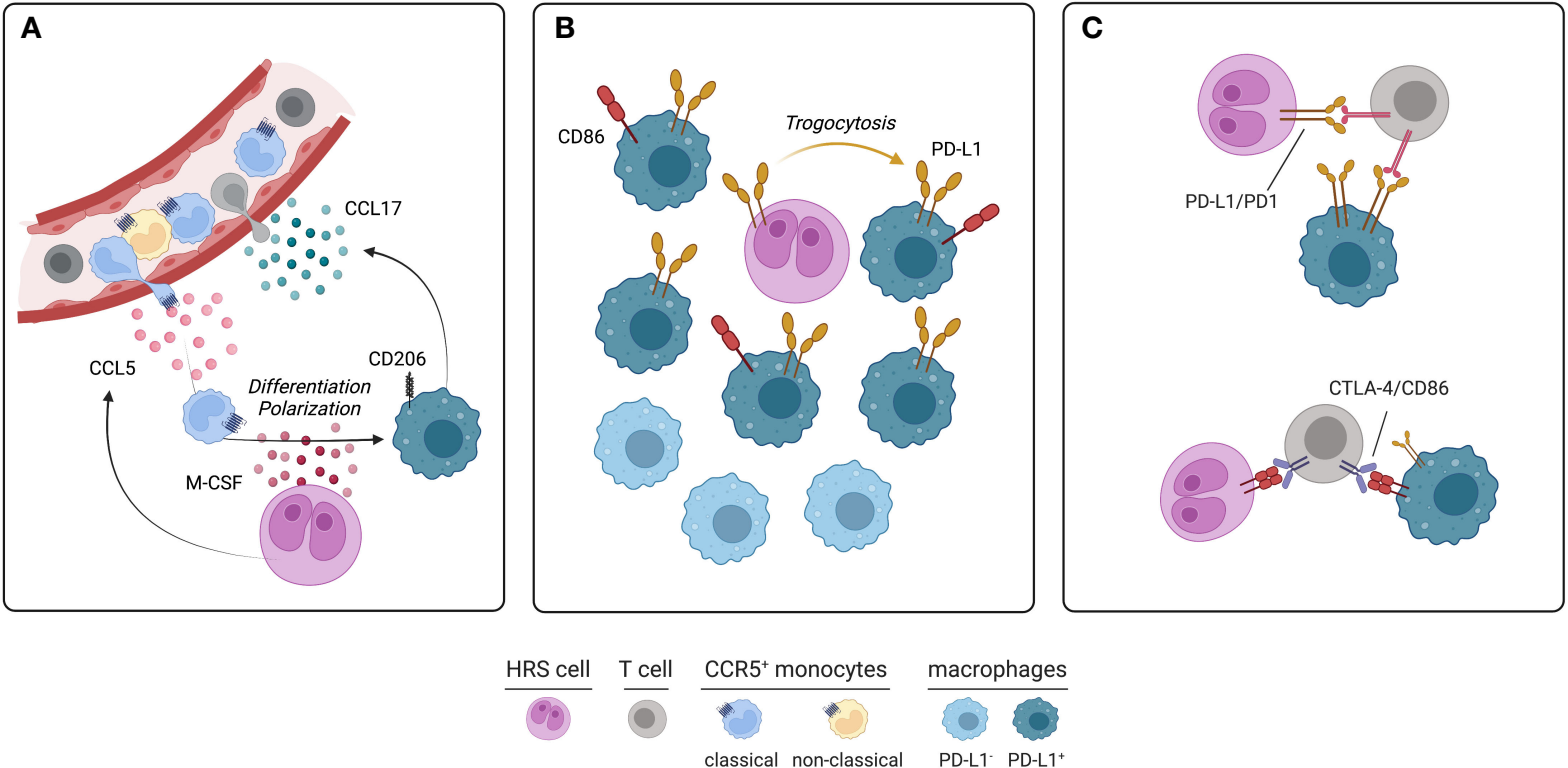
Soluble circuitries, spatial localization and specific interactions of monocyte-macrophages in cHL. **(A)** Circulating CCR5^+^ classical monocytes, which are often more abundant in cHL than healthy subjects, are recruited into tumor microenvironment by CCL5 and other chemokines produced by HRS cells. Recruited monocytes undergo differentiation and polarization into immunosuppressive CD206^+^ TAM due to M-CSF exposure. In turn, TAM produce further chemotactic stimuli, such as CCL17, promoting the recruitment of T-cells, particularly Th2 cells. **(B)** PD-L1^+^ and PD-L1^-^ TAM are two different subsets populating different areas of immune microenvironment. Immunosuppressive, PD-L1^+^ TAM, sometimes co-expressing CD86, lie in close proximity of HRS cells. PD-L1 is acquired by macrophages through trogocytosis. By contrast, PD-L1^-^ TAM are usually located more than 75 μm from HRS cells. **(C)** Specific inhibitory interactions occurring between HRS, T-cells and TAM within tumor microenvironment. PD-1 expressed on T-cells interacts with PD-L1 of HRS cells and TAM. Similarly, CTLA-4 expressed on T-cells interacts with CD86 expressed on HRS cells and a subset of PD-L1^+^ TAM (figure created using BioRender.com).

HRS cells release a variety of chemokines and cytokines that actively recruit monocytes from the cHL microvasculature and contribute to their differentiation and functional polarization. CCL5 secreted by HRS cells recruits CCR5^+^ monocytes and mesenchymal stromal cells involved in fibrosis development (35). CCL5 is produced by cHL cell lines and its expression positively correlates with CD68^+^ macrophage abundance in cHL tissues. The anti-CCR5 therapeutic antibody maraviroc blocks the HRS-mediated recruitment of monocytes, thus decreasing the number of TAM within the cHL microenvironment and curtailing tumor growth in cHL mouse models (35). While HRS cells secrete M-CSF to promote the differentiation of recruited monocytes into macrophages, conditioned medium from cultured TAM increases the size of HRS colonies *in vitro*, highlighting a close pro-survival interplay between the neoplastic and monocytic populations (35). Coculture experiments involving HRS cells and monocytes have demonstrated that monocytes are educated by HRS to synthesize and release immunosuppressive cytokines, such as IL-10 and TGF-β, and chemokines, such as CCL17, which in turn mediate the chemotaxis of pro-tumoral Th2 lymphocytes (8, 36–38) (**Figure 1A**). Elevated serum IL-10 is indeed a predictor of inferior PFS in cHL patients treated with chemotherapy (ABVD regimen) or radiation with curative intent (37, 39). Moreover, soluble factors released by HRS cells induce the surface expression of the regulatory molecules CD206, IDO, and PD-L1 on macrophages, further amplifying the local immune evasion (35). High CD206 promotes mannose-dependent endocytosis, collagen uptake and extracellular matrix remodeling (40). While increased PD-L1 expression on TAM dampens T-cell mediated immunity against HRS cells, it provides part of the rational for the therapeutic use of PD-1 blockade, a strategy of proven success in cHL due to the high dependency of HRS cells on the PD-1/PD-L1 axis (41). Lactic acid produced by neoplastic cells has also been found to polarize macrophages into the M2 phenotype (42). RP6530, an inhibitor of PI3Kγ/δ, impairs the glycolytic metabolism of HRS cells, diminishes lactic acid release into tumor microenvironment, and repolarizes TAM into pro-inflammatory macrophages (42). A phase I trial involving cHL patients treated with RP6530 has shown a significant reduction of circulating myeloid-derived suppressor cells, further suggesting that glycolytic end products could contribute to cHL immunosuppression (42, 43). IL-17, which functions as a chemotactic factor for IL-17RA/RC^+^ monocytes, is an additional soluble player involved in the generation of immune microenvironment. About 40% of cHL cases exhibits an IL-17-enriched milieu, with evidence at both transcriptomic and protein level of IL-17 production by HRS cells and bystander T-cells (7, 44). Soluble CD30, released by HRS cells, promotes the recruitment and polarization of Th17 cells, which in turn might favor the recruitment of circulating monocytes by releasing IL-17 and related cytokines (7).

### Spatial localization and phenotypic features of infiltrating macrophages in cHL

Mass cytometry analysis of cHL suspensions identified two macrophage populations that stained positive for PD-L1, MHCII, IRF4 and CD68, but differed for CD163 expression, perhaps reflecting the M1 (CD163^-^) and M2 (CD163^+^) polarization (45). Multiplex immunofluorescence on cHL tissues further identified a subset of PD-L1-negative macrophages and revealed their topological organization and their relations with HRS cells and infiltrating T-cells (5). In all tested cases, PD-L1^+^ TAM were significantly closer to the PD-L1^+^ HRS cells compared to PD-L1^-^ TAM. Accordingly, the distance between PD-L1^+^ HRS cells and PD-L1^+^ TAM was significantly shorter than the distance between PD-L1^+^ HRS cells and PD-L1^-^ TAM (5). Spatial analyses have also highlighted that PD-L1^+^ TAM were more frequently close to helper and cytotoxic PD-1^+^ T-cells than PD-L1^-^ TAM (5). In addition, multiple subsets of T-cells were observed in direct contact with TAM. Particularly, CD4^+^ T-cells were more frequently in contact with TAM than CD8^+^ T-cells, suggesting that PD-L1^+^ TAM may both promote anti-cancer immunity through the antigen presentation process and favor immunosuppression through the PD-1 signaling pathway (5). Because PD-1^+^ Th1 cells have been identified as a functionally exhausted CD4^+^ T-cell subset within the cHL microenvironment (45), these cell-to-cell interactions involving reactive lymphocytes and macrophages may foster the immune paralysis occurring around HRS cells. Moreover, TAM represent the major source of PD-L1 in cHL microenvironment, thus playing a major role in the creation of the immune-privileged niche that is responsible for HRS survival and expansion. Mechanistically, trogocytosis (i.e., the membrane transfer from one cell to an adjacent cell) has been implicated in transferring PD-L1- and PD-L2-enriched membrane fragments from HRS cells to adjacent TAM by direct contact (46). Indeed, PD-L1^+^ macrophages were more frequent in close proximity, and even in direct contact, to HRS cells, whereas more distant macrophages usually stained negative for PD-L1 (46) (**Figure 1B**). Functional experiments also demonstrated that PD-L1 acquisition through trogocytosis had an inhibitory effect on bystander T-cells in terms of IL-2 and IFN-γ production (46). Therefore, the 9p24 copy number alteration, the genetic hallmark of cHL that enhances the expression of PD-L1 and PD-L2 on malignant cells (47), may ultimately increase the expression of these immune-inhibitory molecules on nearby macrophages and foster regulatory/inhibitory interactions between reactive myeloid and lymphoid cells of the microenvironment. Biochemically, PD-1 engagement by PD-L1^+^ macrophages or HRS cells (**Figure 1C**) leads to phosphorylation of its intracellular tyrosine-based switch motif and recruitment of the phosphatase SHP2, which inhibits ZAP70 and the co-stimulatory molecule CD28 in T cells (48). As a consequence, signaling branches derived from T-cell receptor and CD28, including PI3K/AKT and RAS-MEK-ERK pathways, are attenuated (48). Moreover, reverse signaling *via* PD-L1 can stimulate oxidative phosphorylation, prevent programmed cell death, and favor proliferation of HRS cells *in vitro (* 49). Whether PD-L1 reverse signaling is active in macrophages and how it contributes to their immunosuppressive functions remains unclear. As a further proof of their immunosuppressive role, a subset of TAM also participates to the regulatory CTLA-4/CD86 axis. CTLA-4 is an inhibitory receptor expressed on T-cell surface and is mutually exclusive with PD-1 and LAG-3 (6). In a series of 20 cHL cases, CTLA-4^+^ T-cells were overall more abundant than PD-1^+^ and LAG-3^+^ T-cells within cHL microenvironment (6). The majority of HRS cells stained positive for CD86, indicating that CTLA-4/CD86 interaction may be operational in amplifying immune evasion. Moreover, a minor but clearly detectable fraction of TAM expressed CD86. Such CD86^+^ TAM are more frequently located in close proximity to HRS cells and usually co-express PD-L1 (6) (**Figure 1B, C**). Whether CD86 is acquired by macrophages through trogocytosis or induced by paracrine mechanisms needs further clarification.

### Peripheral blood monocyte signatures in cHL

Mass cytometry evaluation of peripheral blood mononuclear cells has revealed that circulating monocytes are overall more abundant in newly diagnosed cHL patients as compared to healthy subjects (50). Among monocyte subsets, classical monocytes are those showing the major increase in cHL patients, whereas the number of intermediate and non-classical monocytes is comparable in healthy donors and cHL patients (50). Release of M-CSF by HRS cells has been summoned in paraneoplastic monocytosis sometimes observed in cHL patients with advanced disease (31). Several studies have documented a negative correlation between monocyte abundance and cHL prognosis (**Table 1**). Koh and colleagues showed that low lymphocyte-to-monocyte ratios (LMR < 2.9) correlated with higher content of TAM in cHL sections and poorer OS (32). Similarly, in a series of 476 newly diagnosed cHL patients, absolute lymphocyte count/absolute monocyte count ratio (ALC/AMC) was an independent prognostic factor for PFS and OS (33). In patients with advanced-stage cHL, ALC/AMC < 2, > 25% CD68^+^ TAM, and IPS > 2 were identified at multivariate analysis as predictors of poor event-free survival and OS (34). Moreover, patients with negative PET scan after two ABVD cycles displayed inferior outcome if their LMR was < 2, suggesting that peripheral immune landscape could be integrated with interim PET to better define patients’ prognosis (51). In a metanalysis of 8 retrospective studies including a total of 3319 cHL patients, low LMR was associated with poor PFS and OS, further highlighting that LMR might be used as a cheap and useful biomarker with potential application in daily clinical management (52).

### Predictive role of monocyte-macrophages in the era of PD-1 blockade and novel strategies for macrophage targeting

Quantitative and qualitative assessment of circulating monocytes is emerging as a potential tool to predict response to immunotherapy. Patients with fewer circulating classical monocytes tend to have more favorable responses to PD-1 blockade (50). An additional circulating innate subpopulation, possibly of monocytic origin, has been recently identified consisting of cytotoxic CD3^-^CD4^+^CD68^+^GrB^+^ cells, which are also detectable in cHL tumor sections (50). These cells, together with B-cells and mature NK cells, are more prevalent in relapsed/refractory cHL patients who respond favorably to PD-1 blockade (50). Histological evaluation of macrophage features can provide additional predictive information for patients treated with the anti-PD1 antibody nivolumab. In a retrospective study including 61 R/R cHL patients, a low number of M2 (CD163^+^c-maf^+^) macrophages was associated with higher probability of complete response to nivolumab and longer PFS (53). In lymph node samples longitudinally collected before and after nivolumab treatment, depletion of CD68^+^ and CD163^+^ macrophages was observed over time (53). Given the proposed detrimental role of macrophages during PD-1 blockade, novel therapeutic strategies are currently under investigation with the aim of targeting innate cells together with PD-1^+^ T-cells and cancer cells. AFM13 is an innate immune engager that concomitantly targets CD30 on HRS cells and CD16A on NK cells and macrophages. In a phase Ib study involving heavily pre-treated cHL patients, the combination of AFM13 and the anti-PD1 antibody pembrolizumab was well tolerated and led to an overall response rate of 83%, suggesting that dual targeting of innate and acquired immunity within the cHL ecosystem might be highly effective even in multiply relapsed patients. Chimeric antigen receptor T-cell (CAR T) therapy has been proposed as an additional platform to overcome the negative impact of infiltrating macrophages in cHL. Anti-CD123 CAR T-cells have been designed to target both the malignant HRS cells and the surrounding CD123^+^ macrophages in cHL (54). *In vitro* data demonstrate that anti-CD123 CAR T-cells rapidly recognize and kill M2 macrophages by 5 days after the beginning of co-culture (54). Moreover, anti-CD123, but not anti-CD19, CAR T-cells were resistant to the inhibitory effects derived from co-cultured M2 macrophages, thus preserving the ability to secrete pro-inflammatory and anti-tumoral cytokines, such as IFN-γ, TNF-α, and IL-2 (54).

## Concluding remarks

Technical advances in molecular immunology and image analysis have recently revisited the composition and role of infiltrating macrophages and circulating monocytes in cHL. Overall, at least three biological layers are needed to capture the nature of monocyte-macrophages in this disease. The first consists of their surface receptor repertoire, which helps define their differentiation, polarization, and exhaustion-promoting abilities. The second is their secretome, including cytokines and metabolites that recruit further reactive cells and shape the local immune milieu. Third, their typical spatial arrangement, which enables a series of cell-to-cell interactions ultimately leading to HRS cell survival. Although part of this basic science has already been translated into the clinic and contributed to the successful clinical testing of PD-1 blockade, there is still room for therapeutic improvement. A deeper understanding of which receptor/ligand pairs are involved in the inhibitory interactions between macrophages and T-cells could open to novel strategies potentially effective in combination with PD-1 blockade or after its failure. Moreover, prioritizing chemokines that impact the most on monocyte recruitment could reveal new antibody-drug targets to be explored therapeutically.

## Author contributions

IF, AB, EL, AS reviewed the literature. IF and AB wrote the manuscript. MK and CV provide scientific insight and revised the manuscript. All authors contributed to the article and approved the submitted version.
